# A thalamic reticular networking model of consciousness

**DOI:** 10.1186/1742-4682-7-10

**Published:** 2010-03-30

**Authors:** Byoung-Kyong Min

**Affiliations:** 1 Department of Radiology, Brigham and Women's Hospital, Harvard Medical School, 75 Francis Street, Boston, MA 02115, USA

## Abstract

**[Background]:**

It is reasonable to consider the thalamus a primary candidate for the location of consciousness, given that the thalamus has been referred to as the gateway of nearly all sensory inputs to the corresponding cortical areas. Interestingly, in an early stage of brain development, communicative innervations between the dorsal thalamus and telencephalon must pass through the ventral thalamus, the major derivative of which is the thalamic reticular nucleus (TRN). The TRN occupies a striking control position in the brain, sending inhibitory axons back to the thalamus, roughly to the same region where they receive afferents.

**[Hypotheses]:**

The present study hypothesizes that the TRN plays a pivotal role in dynamic attention by controlling thalamocortical synchronization. The TRN is thus viewed as a functional networking filter to regulate conscious perception, which is possibly embedded in thalamocortical networks. Based on the anatomical structures and connections, modality-specific sectors of the TRN and the thalamus appear to be responsible for modality-specific perceptual representation. Furthermore, the coarsely overlapped topographic maps of the TRN appear to be associated with cross-modal or unitary conscious awareness. Throughout the latticework structure of the TRN, conscious perception could be accomplished and elaborated through accumulating intercommunicative processing across the first-order input signal and the higher-order signals from its functionally associated cortices. As the higher-order relay signals run cumulatively through the relevant thalamocortical loops, conscious awareness becomes more refined and sophisticated.

**[Conclusions]:**

I propose that the thalamocortical integrative communication across first- and higher-order information circuits and repeated feedback looping may account for our conscious awareness. This TRN-modulation hypothesis for conscious awareness provides a comprehensive rationale regarding previously reported psychological phenomena and neurological symptoms such as blindsight, neglect, the priming effect, the threshold/duration problem, and TRN-impairment resembling coma. This hypothesis can be tested by neurosurgical investigations of thalamocortical loops via the TRN, while simultaneously evaluating the degree to which conscious perception depends on the severity of impairment in a TRN-modulated network.

## Background

The subjective experience of consciousness is central to our everyday life. However, whether such subjective experiences have neural correlates remains unsolved and open to hypothesis and investigation. For instance, Lamme [1] supported the notion that feedback connections to the primary visual cortex are necessary for visual awareness, and proposed that a progressive build-up of recurrent interactions results in conscious awareness. Dehaene et al. [2], in their 'global workspace' model of consciousness, suggested that conscious perception is systematically associated with parieto-frontal activity, causing top-down amplification. On the other hand, Zeki [3] argued against a single entity of consciousness, claiming that there are multiple hierarchical consciousnesses (the micro-consciousnesses). Therefore, we need a unified theory to integrate these previous theories and provide us with a clearer understanding of all the phenomena of consciousness.

**Figure 1:**
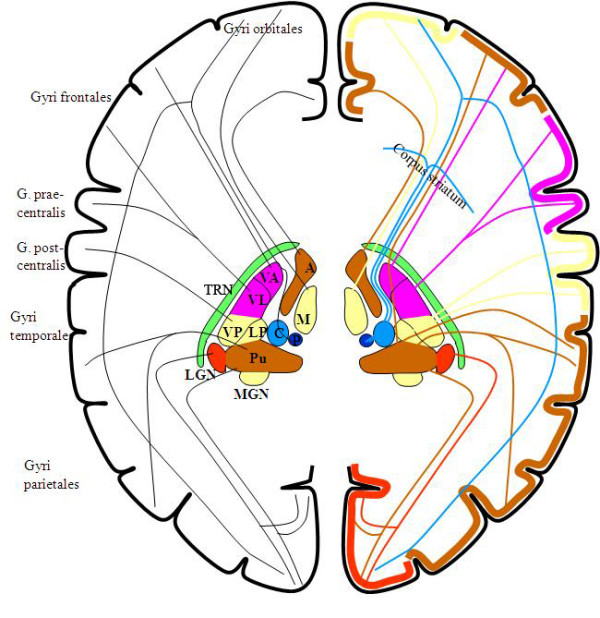
**A schematic diagram of the connections between thalamic relay nuclei and their corresponding cortical areas (of the same color) through the thalamic reticular nucleus**. Black lines indicate corticothalamic connections, and colored lines indicate thalamocortical connections. A: anterior thalamic nucleus, M: medial thalamic nucleus, VA: ventral anterior nucleus, VL: ventral lateral nucleus, VP: ventral posterior nucleus, LP: lateral posterior nucleus, Pu: pulvinar, C: centromedial nucleus, P: parafascicular nucleus, LGN: lateral geniculate nucleus, MGN: medial geniculate nucleus, TRN: thalamic reticular nucleus (courtesy of Wolfgang Klimesch, with permission).

In addition to regarding consciousness as a biological phenomenon, we cannot deny that there is a genuine phenomenon of consciousness in the ordinary sense and that it has distinctive features that should be investigated when seeking to fully characterize it. One of the general agreements is the quality of 'being aware.' Hence, an information-input mechanism can be considered essential to initiate conscious awareness (see Appendix 1), no matter what is evoked inside or outside of the body. It then becomes reasonable to consider the thalamus one of the primary candidates for the seat of consciousness, given that the thalamus has been referred to as the gateway of nearly all sensory inputs to the corresponding cortical areas [4]. As shown in Figure 1, the thalamus is a finely organized neuroanatomical structure with each modality-specific domain sector interconnecting with other corresponding brain structures [5-7]. For instance, the lateral geniculate nucleus (LGN) has reciprocal connections with visual cortices [8,9], and the medial geniculate nucleus (MGN) is anatomically interconnected with auditory cortices [10-12]. In addition, the lateral/medial ventral posterior nuclei are reciprocally connected with primary somatosensory cortices [13,14], while the ventral anterior nuclei receiving afferents from the internal globus pallidus [15] are linked with premotor cortices [7]. All of these anatomical interconnections imply significant functional interconnections; indeed, the thalamus has been regarded as a hub of sensory-motor control.

## Hypotheses

### Consciousness: A mental state embodied through TRN-modulated synchronization of thalamocortical networks

As the cortex has gradually evolved to be competent in higher cognition, the thalamus has evolved in parallel [4]. All the thalamocortical pathways may in fact constitute a unified and cyclic oscillatory circuit that is topographically organized [16]. Interestingly, in an early stage of brain development, communicative innervations between the dorsal thalamus and telencephalon must pass through the ventral thalamus [17], the major component of which is the thalamic reticular nucleus (TRN), a sheer laminar wrapping around the thalamus. It is worth noting that the TRN occupies a striking control position in the brain. The cerebral cortex and thalamus connect in a reciprocal manner, branching onto the TRN as shown in Figure 1. In turn, the TRN sends axons back to the thalamus, roughly to the same region where they receive afferents [18]. The TRN provides a major inhibitory input to thalamic relay nuclei [19]. The inhibitory TRN cells are densely innervated by collaterals from thalamocortical and corticothalamic neurons, both of which generate strong excitatory postsynaptic potentials [20].

Synchronization within a certain neuronal ensemble is achieved by means of GABAergic inhibitory neurons. For instance, it has been reported that neuronal synchronization is completed via a GABAergic internetwork in some pace-making sources such as the suprachiasmatic nuclei [21] and hippocampus [22]. In general, confirming synchronization of asynchronous neural activity is a prerequisite for interpreting these signals as physiologically and functionally associated. Synchrony in the interconnected circuitry of the thalamus and cerebral cortex is particularly critical in conscious events [23]. Therefore, there should be a neural controlling system that regulates globally chaotic neural activities into a unitary conscious entity in terms of synchronization. In the conscious state, the experiences of the internal and external milieu merge into a temporally and spatially unitary experience [24]. McCormick [25] suggested the possibility of a cyclical thalamocortical interaction whose key feature is the strong activation of GABAergic neurons within the thalamus. Taken together, the findings referenced above lead me to hypothesize that the inhibitory TRN cells play a key role in coordinating our conscious perception through the inhibitory feedback network across both the thalamus and the cortex. For instance, it has been suggested that TRN neurons in absence epilepsy may work as a subcortical pacemaker responsible for spike-wave discharges [26,27]. Intriguingly, the TRN shows axons giving off local branches within the nucleus itself [28]. TRN cells are principally coupled via inhibitory GABAergic synapses [29], mostly generating gamma activity [30,31]. Indeed, GABAergic TRN cells demonstrate several frequencies of rhythmic oscillations [32-34]. Some rhythms such as spindle oscillation (7-14 Hz), delta oscillation (3-5 Hz), slow oscillation (0.3-0.5 Hz), and ultraslow rhythm (0.05 Hz) are spontaneously initiated or heavily involved in the TRN [35-38]. It has been suggested that intrinsic pacemaker alpha activity underlies the genesis of spindle waves related to sleep [39]. However, it was found that a large proportion of TRN cells (about 34%) discharged like clocks within a 25-60 Hz frequency bandwidth (i.e., gamma activity) [31].

When a GABAergic network induces synchronization of neural activity, coherent gamma oscillations are observed [40]. The gamma-range (more than about 30 Hz) synchronization is occasionally considered a key mechanism of information processing in neural networks [41,42]. Again, the TRN is located in a particularly suitable position for controlling the entire cerebral network. Therefore, TRN-mediated synchronization in the thalamocortical network may result in gamma-band oscillations related to the binding of the stimulus features into a whole [43,44]. Moreover, cortical gamma activity is concurrent with thalamic gamma activity at discrete conscious events [45]. Most likely, neural synchronization initially driven by the TRN modulates gamma oscillations throughout the thalamocortical loops. Empirically, in an animal study (see Appendix 2), it was proposed that such coherent firing at gamma frequencies reflects a point of equilibrium in the TRN when the leaky K^+ ^conductance is fully suppressed by the metabotropic effects of monoamines or excitatory amino acids [30].

As for more empirical evidence, we may pay attention to the electrophysiological dynamics of the TRN. For instance, I_T _(the T-type current underlying prolonged Ca^2+^-dependent burst firing in GABAergic neurons of rat TRN) of the TRN shows much slower kinetics than do thalamocortical relay cells [46], where I_T _is the critical current for controlling the thalamic response mode [47]. More importantly, I_T _in the TRN needs depolarization for activation. The expression of I_T _depends on its state of inactivation. At hyperpolarized potentials, as during the early stages of sleep, I_T _becomes deinactivated and can produce low-threshold spikes during subsequent depolarization. At sufficiently depolarized potentials, as occur more frequently during wakeful activity, inactivation prevents I_T _conductance [48]. Hence, these different kinetics and specific necessary active conditions between the TRN and thalamic relay cells would critically indicate that interactions between relay cells and the TRN are essential for synchronization [49]. Apparently, if large neural ensembles of the TRN burst rhythmically, their interconnections could facilitate continuance of subsequent synchronous firings. Consistently, bilateral lesion of the rostral pole of the TRN in rats promotes thalamocortical dysrhythmia [50].

Indeed, TRN cells are called the pacemaker for thalamic oscillation [39,47,51-53], and they demonstrate two firing modes: burst-spike and tonic-spike [54]. In relation to the switch mechanism between these two firing modes, Mistry et al. [55] reported frequency-dependent short-term modulation at glutamatergic synapses in the TRN. They found that TRN neurons exhibited no short-term change in alpha-amino-3-hydroxy-5-methylisoxazole-4-propionic acid (AMPA) receptor-mediated excitatory postsynaptic current amplitudes in response to stimulation at non-gamma frequencies (less than 30 Hz), simulating background activity, but showed short-term depression in such amplitudes at gamma frequencies (more than 30 Hz), simulating sensory transmission [55]. The same study also found that intra-TRN inhibition suppresses TRN tonic-spike selectively at non-gamma stimulus frequencies, which are indicative of background activity. Presumably, in the absence of sensory transmission, the intra-TRN inhibitory network controls the number of spikes fired by TRN cells, consequently regulating the degree of inhibition exerted by the TRN cells onto thalamocortical networks [55]. Therefore, switching between two ranges of stimulus frequencies to TRN cells (gamma and non-gamma range) regulates two physiological modes of TRN cells in the control of TRN output. This switch mechanism, in the TRN-mediated model, may play a gating role in progress to conscious awareness.

As mentioned in the Background section, the thalamus is not a simple relay station in sensory signal processing but is instead involved in many dynamic processes that significantly alter the nature of the information relayed to the cortex [56]. Neurons in the thalamic relay nuclei [57,58] and the TRN [48,59] fire in two activity modes (tonic and burst) as mentioned above. From the viewpoint of a gate-keeping state of the thalamus, tonic mode firing in the thalamus may be responsible for a thalamic-gate passive mode (unconscious state), whereas burst firing may account for a thalamic-gate active mode (conscious state) [60-62]. In keeping with such a gate-keeping mechanism, I hypothesize that a conscious state would be established when a TRN-modulated thalamocortical network activates over a certain threshold to initiate overall synchronization. In contrast, in the sub-threshold state, sensory inputs may simply pass through the thalamus without the generation of conscious awareness. In other words, the brain might actually receive such unconscious sensory inputs, but those signals fail to reach the level of conscious awareness. This interpretation is applicable to the case of implicit knowledge, which is revealed in task performance without any corresponding phenomenal awareness [63].

Furthermore, the main part of the TRN can be divided into functionally distinct 'sectors' on the basis of its afferent connections with groups of thalamic nuclei and cytoarchitectonically definable cortical areas [4]. Therefore, there are relatively accurate topographic maps corresponding to the same modality in the TRN [64,65]; these cumulative maps of the latticework [66] appear to indicate a nexus, functionally related to the thalamo-cortico-thalamic pathways [67]. More significantly, the lack of clearly definable borders of the sectors as well as a larger receptive field of the TRN [68] suggest that the TRN may be associated with integrative information processing and even cross-modal overlapping awareness. The experience of a unitary consciousness is plausible from the viewpoint of the synchronization of crudely overlapped receptive maps on the laminar TRN. Similarly, we can gain a comprehensible sense of the modal-confused symptom in which unconscious priming eliminates the automatic binding of color and alphanumeric form in synaesthesia [69]. Within the framework of a TRN-synchronizing model, a variety of overlapping combinations of neural ensembles for conscious perception are plausible. These types of patterns of neural combinations for conscious awareness may lead to intra-individual variation in conscious perception in terms of 'qualia' and inter-individual 'subjectivity' in experiencing consciousness. I will discuss the topic of conscious awareness in more detail in the following setion for 'awareness'.

Taken together, evidence thus far suggests that the TRN is central in determining the initiation of communicative interactions between the thalamic relay nuclei and the cerebral cortex. Consequently, it likely plays a key role in controlling our unitary conscious perception. Therefore, the feedback synaptic connections from the TRN imply its potentially significant role in modulating the transmission of information in the thalamocortical circuit.

Additionally, to sustain a conscious state, arousal is necessary for the threshold condition of the TRN. Surely there are anatomical connections between the TRN and brainstem cells that control the subject's wakefulness and vigilance [47]. Moreover, an inhibitory influence on the activity of TRN neurons is exerted by threshold stimulation of the mesencephalic reticular formation (MRF), which is the core of the brainstem, while supra-threshold stimulation of the MRF induces the activation of TRN neurons [70]. Those researchers concluded that the synchronizing structure of the brainstem, exerting a blocking impact on the MRF, facilitates the activity of TRN neurons. It is also reported that the brainstem has relatively uniform effects on the response mode of relay cells throughout the thalamus [71]. As mentioned earlier, the response mode of thalamic relay cells is principally under the command of the TRN, so these findings are still comprehensible within the framework of the TRN-mediated conception. Taken together, previous findings indicate that the brainstem exerts substantial control over the activity of TRN neurons, possibly to globally modulate the level of arousal for preparing for consciousness.

Besides, since consciousness is considered a biological phenomenon, gene polymorphism undoubtedly contributes to fine variations in consciousness among individuals. For example, since the TRN-mediated consciousness network principally involves GABAergic synapses, polymorphisms in several GABA-related genes have been associated with differences in the efficiency of mental processing [72-75]. Furthermore, AVPR1a and SLC6A4 gene polymorphisms have been reported in association with creative dance performance, which can be related to altered consciousness states [76-78]. Indeed, consciousness is completed through many neuronal assemblies, so it is substantially subject to diversity in genetic expression. However, the genetic effects on consciousness seem to be relatively modest, since they interact with environment or experience during the development of the network.

### Attention: Highlighted thalamocortical synchronous activity coordinated by the TRN and associated cortical areas

**Figure 2:**
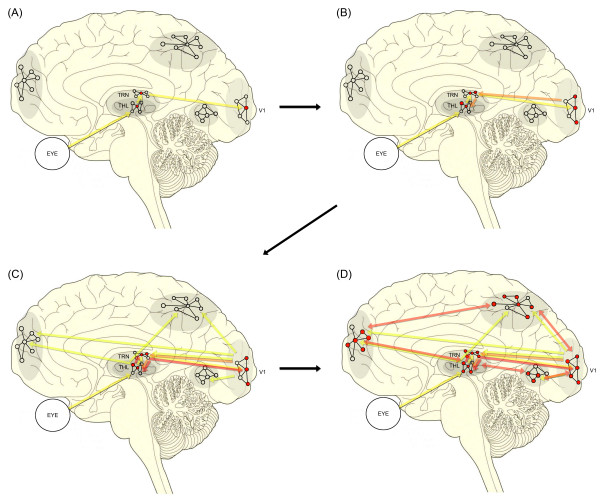
**Schematic drawing of the TRN-modulated thalamocortical looping model of conscious awareness**. THL: thalamus, V1: the primary visual cortex. As the color of processing-flow lines gets darker (from yellow to orange, and finally to red), more elaborated information processing is being produced by means of iterating thalamocortical loops through the TRN. Cortical networks tie together neuronal assemblies in widespread cortical regions, and the TRN may play a central role in organizing all of the networks.

Attention possibly acts by biasing the competition among rival candidates of activated neuronal sets, particularly during their formation [79]. Therefore, an antagonistic target-background configuration of information processing seems to be an efficient means to accomplish selective attention. That is, it is advantageous if there is enhancement of neural activity associated with highlighted information processing, while irrelevant neural activity is simultaneously inhibited. Therefore, an efficient and well-organized inhibitory mechanism is necessary for selective attention. As mentioned earlier, it is noteworthy that the TRN provides a major inhibitory input to thalamic relay nuclei [19]. Hence, the feedback synaptic connections from the TRN imply significant control over the signal transmissions through the thalamocortical network. Anatomically, specified sectors of the TRN involve their corresponding thalamocortical connections, including even the related visceral sensory inputs [80], which are assumed to be modulated by attention or distraction. Here, I suggest that such finely organized TRN cells, wrapping around many of the thalamic relay nuclei, play a pivotal role in selective attention by coordinating all thalamocortical transmissions via the GABAergic network synchrony. The initial point of developing synchronization may stem from a modality-specific sector of the thalamic reticular region (e.g., the first red dot on the TRN in Figure 2A), which may be the origin of the eventual spread of its synchronous activity throughout the entire TRN network. This synchronization would be more promoted after the reception of positive iterating feedback from higher-order cortices. Compared to Crick's previous TRN hypothesis regarding 'attention' [81], my current TRN hypotheses also cover consciousness, and emphasize reiterating thalamocortical information processing in relation to conscious awareness.

As illustrated in Figure 2, the TRN-modulation model is compatible with models of large-scale cortical networks [82,83] and of parallel distributed processing [84]. Within these frameworks, cognitive functions are widely distributed in cortical networks and processed with basic features such as parallel input-to-output connections and reentry or recurrence, which can occur within and between layers of the cortical hierarchies. It has been suggested that the operation of a large-scale network in cognitive function is principally based on the correlation of the firing of its elements across cortical regions [82,85,86], which is the neural manifestation of 'binding' [24]. Therefore, such reentry processes propagating over all of the brain networks are not static but dynamic, which is probably what William James called 'the stream of consciousness' [87].

Indeed, there is substantial evidence that cortical areas play significant roles in attention and cognition processing. For example, the prefrontal cortex, at the top of the executive hierarchy, is critical in decision-making [88-90]. As it plays a pivotal role in attention and working memory, the prefrontal cortex has occasionally been considered the seat of consciousness [24]. As long as the prefrontal cortex controls cognition, its role in consciousness is obviously important. This is particularly the case in the attentive processes that lead a pattern of behavior, speech, or reasoning to its goal [24]. Among those processes, working memory is the most closely engaged with consciousness in the temporal domain. This is because the prefrontal cortex houses the process of temporal integration, which makes possible the persistence of cognitive content.

Although the prefrontal cortex receives an immense quantity of afferent influences from the rest of the brain, the majority of these influences are, nevertheless, sometimes processed out of consciousness. For example, the prefrontal cortex mediates unconsciously triggered inhibitory control in the Go/No-Go paradigm [91]. Thus, the cortex seems to play a subsidiary role in conscious awareness, either when selecting the information upon which one focuses or when directing the whole neural network involved in information processing. Presumably, the degree and distribution of cortical activity involved in cognition determine the content of consciousness [24].

However, compared to the functional roles of individually distributed cortices, the TRN seems to play a critical and supervising role in controlling the whole brain network. Attention is eventually accomplished through cooperatively integrating information from attention-related cortical regions (e.g., the dorsolateral prefrontal cortex [92,93], the parietal cortex [94-97], and the orbitofrontal cortex [98,99]) and from other sub-cortical regions such as the superior colliculus. In this respect, the inhibitory feedback mechanism of the TRN on the thalamocortical network becomes a potential candidate for controlling and coordinating the orientation of attention. In accordance with this conception, TRN lesions effectively prevented perseverative behavior in rats, while lesions of the orbitofrontal cortex failed to do so [100].

Several behavioral phenomena regarding attention are comprehensible within the framework of the TRN model. In 'change blindness' experiments, normal observers are thoroughly unaware of an essential part of what is going on in their visual field. One possible mechanism underlying the lack of awareness for events from which attention has been exogenously diverted could be a defective integration of elementary features for these events [101]. This interpretation is understandable from the viewpoint of the integrative and mastery role of the TRN in attention to accomplish complete awareness. Another phenomenon related to exogenous attentional orienting is 'inhibition of return' (IOR) [102,103]. With reference to the need for responding to novelty while resisting distraction, exogenous orienting processes are good candidates for involvement in drawing attention to novel events [104] by inhibiting repeated orientations toward the same locations (i.e., IOR). As mentioned earlier, inhibitory control is the crucial feature of the internal dynamics of the TRN model, allowing it to suppress interference effects and promote the exploration of the visual scene. In addition, to successfully cope with a continuously changing environment, efficient mechanisms for 'change-detection' are necessary. A recent study provides experimental evidence that the TRN neurons of the rat respond more strongly to deviant stimuli than standard stimuli, whereas the medial geniculate nucleus showed such deviance detection on a smaller scale [105]. Therefore, the TRN seems to work more dynamically to detect changes than thalamic relay neurons do. Probably, consistent thalamocortical feedforward-feedback processes that act on pre-existing neural representations on the TRN interfere with new incoming signals, eventually competently detecting changes.

From the viewpoint of the TRN model, the Hebbian neural network [106] can be conceived, for example, in terms of the object-based neural connection for selective attention [107]. Locally and transiently synchronized neural ensembles, possibly evoked by each tentative object (or mental target), could be seen as candidates for a dominant signal that controls the working memory domain (see Appendix 3). Therefore, if one of these object-based neural candidates is selected ('highlighted') as a mental target, we may conventionally refer to this neurophysiological phenomenon as 'paying attention.' Thereby, such multiple activated neural candidates can be analogous to what Zeki called the micro-consciousness [3]. This interpretation is also consistent with Dehaene's conception of pre-conscious processing [2]. Taking into consideration both the TRN-mediated model and the Hebbian cell assembly, substantial synchronization in the TRN would not always be necessary. In other words, if the input information is relatively new to the thalamocortical networks, substantial synchronization in the TRN is explicitly expected. In contrast, for relatively familiar signals, the Hebbian network, already established in the relevant thalamocortical tracks, may facilitate subsequent processes, which would effectively diminish coherent synchronization compared to what is expected for new signals. Therefore, it is probable that TRN-modulated thalamocortical synchronization depends on the familiarity of signals and that the TRN-modulation model is consistent with the Hebbian model. Moreover, it is likely that 'working memory' can be thought of as temporal mental traces of attended conscious awareness during a transient time range around the present. The circulating model explains feasibly how working memory is developed by any related attention. Presumably, working memory is a type of transient active mental tracing network, which is cued by initial looping signals reiterating over their correspondent thalamocortical circuits. Therefore, these neural traces should be critically related to the property of initial signals for the corresponding thalamocortical circuit. Consistently, such an associative property is one of the characteristics of working memory.

Through this TRN-modulation model, the concepts of capacity restriction of attention and the inability of humans to carry on simultaneous multi-attention become understandable. The limited capacity of attention may be caused by mechanical limitations in synchronizing TRN activities as a whole. Finally, such synchronization should yield a dominant single output signal as a unique mental representation related to the attended object. Reasonably, this characteristic of restrictive attention is more severe within the same modality, as the input information of each modality belongs to each corresponding sector in the thalamic relay nuclei as well as in the TRN. Therefore, the finely compartmental configuration of both the thalamus and the TRN is subjected to exclusive competition for the same modal signals. Apparently, this hypothesis is also consistent with bi-stable perception, unilateral neglect, extinction, and simultagnosia, because the TRN in each hemisphere has no direct connection between them. Hence, they are able to exert efficient lateral control over each hemispheric thalamocortical loop. However, ultimately they should communicate indirectly to result in a unitary conscious awareness, which may represent somewhat unclear emotional conscious experiences, such as a melancholy mood. Consistently, there is a connection between the TRN and the limbic system [66] that is related to emotion. Moreover, the phenomenon of 'attentional blink' appears to be in accord with this TRN-synchronizing model, as the TRN network would need an absolute refractory period in which to switch modes of synchronization in order to shift attention to other upcoming targets. Therefore, such a refractory period in changing attention could account for 'attentional blink'.

In addition, the unilateral characteristic of attention is comprehensible to a certain extent through a number of TRN-lesion studies. For instance, reduced right tecto-pulvinar activity was offset by over-compensatory enhancement in the TRN suppression of left pulvinar activity [108]. Unilateral electrolytic lesions of the TRN elicited metabolic depression in the ipsilateral thalamic centrolateral, mediodorsal, ventromedial, and ventrolateral nuclei, and metabolic activation in the bilateral dorsal tegmental nuclei [109]; and a selective excitotoxic lesion of the ipsilateral TRN induced changes in the receptive field properties of the contralateral vental posterior medial thalamic nuclei [110]. Furthermore, even in the study of memory impairment, the unilateral inactivation of rats' TRN has been shown to interfere with the acquisition of active avoidance in the contralateral hemisphere [111]. Therefore, the gating role of the TRN in the information flow between thalamus and cortex seems plausible in a unilateral manner, and these anatomical and physiological features may lead to the unilateral characteristic of attention.

### Awareness: Conscious perception of an attended mental representation by strengthening relevant neural networks through thalamocortical reiterating

It is important to note that, although attention seems to be a necessary condition for awareness, it appears by no means to be sufficient. For example, exogenous cues presented below a subjective threshold of awareness can capture attention automatically but without awareness [112]. Therefore, we need to identify more complementary mechanisms underlying conscious awareness. Regarding the thalamocortical feedback mechanism, Sherman and Guillery [67] reported that first-order thalamic relay cells receive their driving afferents from ascending pathways and send these signals to their corresponding cortices for the first time, whereas higher-order thalamic relay cells are held to bring their principal messages from the corresponding cortices. Interestingly, TRN cells send their axons back to the thalamus, particularly two branches of a single axon connected to the first-order and the related higher-order nucleus [113]. Thus, the TRN can be said to act as an integrative junction of different but associated thalamocortical circuits. Sherman and Guillery [67] suggested that the functional significance of such a gathering venue may be most important for the interactions among first-order and higher-order circuits that belong to the same modality grouping.

Based on such anatomical evidence, the present study hypothesizes that conscious awareness may be embodied in the process of such thalamocortical iterative signal circulation as controlled by the TRN, as depicted in Figure 2. To elaborate the degree of conscious awareness, first-order and higher-order relay cells may interact within the TRN, where the closest relationships between first-order and higher-order relay circuits for any one modality are found [67]. Accordingly, the first-order and higher-order relay circuits controlled by the TRN can yield more refined and thus higher cognitive information, as their circulating feedbacks run over and over again in an integrative reprocessing manner. In this sense, the compact latticework formation of the TRN is advantageous to coordinate the overall conscious experience.

This dynamically circulating model offers one plausible understanding of the typical characteristics of consciousness. For example, although consciousness appears to be continuous, it actually consists of isolated steps [114,115]. According to the TRN-modulation model, consciousness consists of each mental unit, which is an individual thalamocortical looping mechanism, no matter what cognitive stages it involves. Such individual mental units may behave discordantly, possibly reflected in the discrete characteristic of consciousness. In addition, as we experience everyday life, we require at least a minimal period to become aware of something. Indeed, in the framework of the TRN-mediated thalamocortical looping model, minimal time is necessary for signals to loop the circuit. This model is also in accordance with the recently proposed hypothesis that we are only aware of changes in our underlying cognition [116]; according to the TRN-modulation model, only dynamically iterating neural signals through thalamocortical loops eventually substantiate our conscious perception.

In line with the plausible account of characteristics of consciousness, the mechanism of dreaming is also explainable within the framework of this circulating model. In spite of nearly complete lack of sensory input from the real world during sleep, the looping circuit of the TRN-centered circulating model could be activated by randomly evoked signals within that loop, which presumably initiates to evoke a dream mechanism. Therefore, during dreaming we can have mental experiences as vivid as those we have in the real world. Similarly, this circulating model is consistent with 'self-consciousness,' because the model allows thalamocortical networks to generate signals for themselves merely by an internally driven cue such as visual imagination (with the eyes closed). This circulating model can explain the priming effect as well. Possibly, a priming signal may boost or initiate the related neural activation of the TRN-mediated thalamocortical network in advance so that a subsequent relevant input signal could advantageously advance this warmed-up activation into more proliferated activation. The priming effect appears therefore to be modality-specific [117], which is also a particular structural characteristic of the TRN, as mentioned earlier.

## Conclusions

Thus far, I have argued that the TRN-modulated thalamocortical network appears to have evolved as the neural correlate for conscious awareness. Presumably, the conscious state is a TRN-modulated synchronized neural state that enables us to facilitate subsequent attention processes. In striking accordance with these hypotheses, if the TRN is somehow impaired, the main symptom resembles coma (akinetic mutism) [118]. The TRN may serve as an action coordinator for controlling our selective attention and subsequently for filtering irrelevant signal processing in conscious perception of a target mental representation. The TRN most likely screens perceptual representation, particularly through its modality-specific sectors. The coarse boundary configuration of representing maps in the TRN likely enables us to experience conscious awareness as a complete entity, which I refer to as unitary conscious awareness. I presume that the framework of conscious awareness is a reiterate circuit looping over the thalamus-TRN-cortex, where the TRN would control a gate-keeping mechanism for attention. Higher cognitive processes are likely to be achieved through such repeated thalamocortical looping. I expect that both top-down and bottom-up signals can communicate in the thalamocortical looping circuits and thus create a finally unified percept by their synchrony, which is mediated substantially by the TRN. To sum up, the TRN may work as a gatekeeper for consciousness and as a communicative screen for conscious awareness. The TRN-modulation hypotheses for consciousness, attention, and awareness can be summarized as follows:

[1] 'Consciousness' is referred to as thalamocortical response modes controlled by the TRN and is embodied in the form of dynamically synchronized thalamocortical networks ready for upcoming attentional processes.

[2] 'Attention' is neurophysiologically substantiated by a highlighted neural ensemble among a number of synchronized thalamocortical candidates, the topographical maps of which are projected onto the TRN.

[3] Thalamocortical looping via the TRN is necessary for the 'conscious awareness' of an attended object.

We can test these hypotheses experimentally. For example, the degree of conscious awareness could be evaluated along with the severity of the impairment of the TRN. We can also assess the degree of modality-specific conscious perception while investigating the extent of impairment in its functionally relevant thalamocortical loops. The influence of neuroanatomical abnormal connections (whether they are on the first-order track or on higher-order networks) could also tell us more about the significance of iterative thalamocortical loops in accomplishing improved conscious perception. Most likely, the phenomenon of masking [114] presents compelling evidence for the significance of thalamocortical loops in conscious perception. Masking phenomena can be interpreted as an interruption between the signals circulating through the thalamocortical loops.

The TRN-mediated model could reconcile several major hypotheses for conscious awareness. According to the so-called 'global workspace' model of consciousness [2], incoming information becomes conscious only if three conditions are met: (1) the existence of represented information by sensory neural networks; (2) the existence of representation long-lasting enough to gain access to a second stage of processing; and (3) the ignition of a combination of bottom-up propagation and top-down amplification to create a state of reverberating, coherent activity. The first condition of the global workspace model is well in accordance with the networking conception of the TRN-mediated model. The second condition of the global workspace model can be understood from the viewpoint of the thalamocortical iterative looping of the TRN-mediated model. Likewise, the third condition of the global workspace model is analogous to the aspect of synchronized neural activation in the TRN-mediated model.

Compared to the global workspace model, the TRN-mediated model seems more plausible in the following respects. The model-specific sectors on the TRN provide us with a deeper understanding of modality-specific conscious awareness. In addition, although the prefrontal cortex is an important center for conscious awareness in the global workspace model generally, the prefrontal cortex is sometimes not necessary to produce a kind of modality-specific awareness in the TRN-mediated model. Based on the TRN-mediated model, the TRN is responsible for controlling thalamocortical networks to be consciously processed, and the prefrontal cortex may work for the conscious evaluation of highlighted thalamocortical networks.

As shown in Figure 2, the concept of locally synchronized loops such as in the occipital, frontal, or parietal region is consistent in all the three models (Dahaene's, Lamme's, and the present TRN model). These locally activating networks indicate either corticocortical regional networks or any subunit of TRN-mediated thalamocortical assemblies. On the other hand, the distributed firing patterns in the global workspace model [2] are characteristic of the processing stage, when the TRN is initiating to globally control and organize all the locally activating neural networks to bring about the completion of conscious awareness. That is, the TRN invigorates a specific neural ensemble to be consciously processed and simultaneously suppresses other irrelevant neural activations.

In particular, as Lamme [1] emphasized the importance of feedback connections to the primary visual cortex in terms of visual awareness, the TRN model also highlights the significance of feedback signals from the primary sensory cortex to accomplish conscious awareness. Furthermore, Lamme and Roelfsema [119] proposed the necessity of recurrent processing in relation to consciousness. This notion is exactly what the TRN-mediated model has highlighted as iterative looping processes for conscious awareness.

As discussed so far, the TRN model does not underestimate the significance of cortical roles in conscious awareness. It simply highlights the physiological significance of the central roles of the TRN, such as controlling, screening, and accumulating neural signals through thalamocortical loops during conscious awareness. Most likely, the cortical layers communicate regularly through their corticocortical connections [120]. In addition, the cortices provide their own substantial contribution to the development of consciousness. That is, they may elaborate not-yet-matured information into highly processed information while managing previous information held in a certain form in the cortical network. For example, the dorsolateral prefrontal cortex is assumed to be the neural correlate for integrating discrete cycles of conscious perception [116,121]. However, the TRN-modulation model of consciousness can account for more mental experiences while including corticocortical communication within its framework; they are not mutually exclusive. Through the TRN-modulation model, it becomes easier to understand why there are such complicated (seemingly randomized but logically associated) topographic distributions across many cortical areas during conscious awareness. The receptive maps projected on each sector of the TRN are overlapped and may be intercommunicative within the latticework structure of the TRN. Moreover, 'blindsight' phenomena [122] seem less paradoxical from the viewpoint of the TRN-modulation model; with blindsight, the TRN and the thalamus cannot receive any feedback signals from the V1 (primary visual cortex). Such signals from the primary sensory cortex are necessary to accomplish conscious awareness according to the TRN-modulation model. In other words, visual thalamic nuclei such as the LGN or pulvinar would passively receive continuous input from the retina, but without returning signals from the V1, resulting in unconscious perception.

In addition, the possibility that thalamic relay mixtures such as intralaminar or midline thalamic nuclei play a role in integrating information processes for consciousness cannot be excluded, as expected in the TRN. However, compared to the TRN, they appear to make different contributions, because they are connected principally with the striatum apart from the cerebral cortex [123]. Furthermore, the striatum is connected to the TRN in an unusual manner, such that the external segment of the globus pallidus projects onto the TRN without branching on the motor thalamus [124]. Moreover, there is no innervation to the TRN from the internal segment of the globus pallidus, which projects onto the thalamus [125]. These findings imply that the thalamus and the TRN are regulated differently in terms of motor control. Presumably, the consciousness of voluntary movement and that of sensory processing are different in their neural correlates. Indeed, since the prefrontal cortex receives afferents from thalamus, hypothalamus, limbic structures, and brainstem [126], presumably conveying information about motivation and intention of movement, and influences movement only indirectly through projections to basal ganglia, secondary motor cortices, and cerebellum [127], the prefrontal cortex does not seem to be the center of willful motion, but is rather considered as the temporal and executive organization of willful motion [24]. Therefore, we need a more plausible neural controller for initiating and supervising the consciousness of voluntary movement, irrespective of whether it is a whole neural network or a small neural assembly. Given the issue of motion free-will and the remaining questions regarding the functions of thalamic relay mixtures or higher-order relay circuits, further development of the TRN-mediated consciousness model requires further experimental investigation. For example, the interactive relationship between the TRN and higher-order relay cells may be a key feature for screening mental representations to be selected as an attended target and consequently for manifesting conscious awareness of it, as both the TRN and higher-order thalamic relay cells receive feedback signals from the corresponding cortices, where more elaborated information might be uploaded and kept. This crucial relationship requires clarification in further studies. In spite of these remaining challenges, the sophisticated but logically connected thalamocortical circuits passing through the TRN and its inhibitory control function continue to reinforce the significance of the TRN-mediating model of consciousness.

## Appendix

1. To clarify the descriptions used throughout this paper, I tentatively use the term 'consciousness' as a general mental state of being capable of awareness. In addition, the term 'awareness' is used to represent a specific mental state of being acquainted with something in a conscious manner (conscious perception).

2. Some empirical evidence regarding the neurophysiology of the TRN used in this paper is based on animal studies; hence, there are limitations regarding the generalization of the TRN-modulation hypothesis of human consciousness. However, as the human TRN might have evolved from the TRN of animals, I believe that such implications concerning our consciousness have considerable value for future studies.

3. It is another question as to which brain region is responsible for deciding what to attend to. This is perhaps controlled by top-down signals from higher cognitive stages (e.g., the frontal cortex). This issue remains controversial and open to investigation.

## Competing interests

The author declares that the research was conducted in the absence of any commercial or financial relationships that could be construed as a potential conflict of interest.
